# What the papers say

**DOI:** 10.1093/jhps/hny003

**Published:** 2018-02-05

**Authors:** Ajay Malviya

**Affiliations:** Consultant Orthopaedic Surgeon - Northumbria Healthcare NHS Foundation Trust, Senior Lecturer, Regenerative Medicine - ICM, Newcastle University, 10 East Brunton Wynd, Newcastle upon Tyne, NE13 7BR, UK.

The *Journal of Hip Preservation Surgery* (*JHPS*) is not the only place where work in the field of hip preservation may be published. Although our aim is to offer the best of the best, we continue to be fascinated by work that finds its way into journals other than our own. There is much to learn from it so *JHPS* has selected six recent and topical subjects for those who seek a summary of what is taking place in our ever-fascinating world of hip preservation. What you see here are the mildly edited abstracts of the original articles, to give them what *JHPS* hopes is a more readable feel. If you are pushed for time, what follows should take you no more than 10 min to read. So here goes …

## IS SURGICAL VOLUME A RISK FACTOR FOR REVISION SURGERY OR HIP REPLACEMENT AFTER HIP ARTHROSCOPY?

Kester *et al*. [1], from the Hospital for Joint Diseases, New York queried the Statewide Planning and Research Cooperative System database on New York from 2011 through 2012 and identified 3957 patients who underwent hip arthroscopy. They aimed to explore independent risk factors for failure of surgery and divided surgical volume into tertiles for the purposes of statistical analysis. All patients aged 18 years or older who underwent hip arthroscopy according to Current Procedural Terminology coding were included. Longitudinal analysis for a minimum of 2 years was performed to determine risk factors for revision surgery or conversion to total hip replacement (THR).

The mean age was 35.8 years and after a minimum follow-up period of 2 years, the overall failure rate was 9.6%: 3.7% of patients underwent revision hip arthroscopy at an average of 15.8 months, whereas 5.9% underwent conversion to THR at 14.7 months. Index surgery performed by surgeons in the third tertile of surgical volume (<40 cases per annum) was an independent risk factor for revision [odds ratio (OR) 1.71; *P* = .001], as well as conversion to THA (OR 1.90; *P* < .001). Female patients (OR 1.8; *P* < .001), older patients (OR 3.4; *P* < .001) and patients with a history of obesity (OR 5.6; *P* < .001) underwent conversion to THA at significantly higher rates than other patients. Young patients (OR 4.4; *P* < .001) and female patients (OR 1.6; *P* < .001) were more likely to undergo revision hip arthroscopy.

**T**he analysis of 3957 patients found that female sex, age under 40 years, absence of a labral repair and index procedure performed by a low-volume surgeon were independent risk factors for revision hip arthroscopy. Age over 60 years, index procedure performed by a low-volume surgeon, female sex, obesity and the presence of pre-existing arthritis were risk factors for THA conversion.

## WHAT HIP PRESERVATION OPTIONS ARE EFFECTIVE FOR AVASCULAR NECROSIS OF THE FEMORAL HEAD?

Yu *et al*. [2] from Zhejiang, China have performed a meta-analysis of randomised controlled trials on the effectiveness of various hip preservation treatments for non-traumatic osteonecrosis of the femoral head.

The primary outcome measure was treatment failure rate and secondary outcomes included the Harris hip and the Western Ontario and McMaster Universities Osteoarthritis Index (WOMAC) scores. The search identified twenty-one articles assessing a total of 1415 hips in our analysis. In the network meta-analysis, the treatments were ranked by the surface under the cumulative ranking curve (SUCRA). Core decompression (CD) plus cytotherapy was most likely to reduce the treatment failure rate (SUCRA score = 18.9%), followed by alendronate treatment (SUCRA score = 17.8%), cocktail treatments (SUCRA score = 15.6%), extracorporeal shock wave therapy (ESWT) plus alendronate (SUCRA score = 15.4%) and avascular biomaterials plus cytotherapy (SUCRA score = 13.8%) in a frequentist framework; similar results were obtained in a Bayesian framework. For the secondary outcomes, ESWT was most likely to improve the Harris hip score (SUCRA score = 33.7%), followed by ESWT plus alendronate (SUCRA score = 33.1%) and cocktail (SUCRA score = 19.6%) treatments in a frequentist framework. A traditional analysis showed that the effect of CD plus cytotherapy was significantly better than the effect of CD alone in improving the WOMAC score (SMD −6.01; *P* < 0.001).

CD plus cytotherapy is a relatively superior treatment for reducing treatment failure rates in early and intermediate ONFH patients, and ESWT is the most effective treatment for improving Harris hip scores.

## DOES RESECTION OF A POSTEROSUPERIOR CAM LESION DECREASE THE FEMORAL HEAD PERFUSION?

Disruption of the arterial supply to the femoral head, and subsequent development of femoral head osteonecrosis, is of serious concern with intracapsular hip procedures. However, the effect of arthroscopic femoral osteochondroplasty on femoral head perfusion is unclear. Lazaro *et al*. [3] aimed to quantify the effects of both standard and posterosuperior extension of arthroscopic femoral osteochondroplasty on femoral head vascularity. They hypothesised that extension of the superior resection zone posteriorly would negatively affect femoral head perfusion.

In 12 cadaver pelvic specimens, the authors cannulated the medial femoral circumflex artery (MFCA). One hip per pelvis was randomly selected to be in one of two experimental groups based on the superior extent of the osteochondroplasty: standard resection [resection anterior to the 12 o'clock (0° of 360°) position] or extended resection (resection extended posterior to the 12 o'clock position). Computed tomography scans were obtained prior to and following arthroscopic resection to delineate the resection margins. Gadolinium enhancement on magnetic resonance imaging (MRI) was quantified in the femoral head by volumetric analysis using custom software. A polyurethane compound was injected and gross dissection of the vasculature was performed.

Extension of the osteochondroplasty posteriorly (the extended-resection group), to a mean of 41.3° (range 34–47°) posterior to the 12 o'clock position, decreased femoral head perfusion by a mean of 28% (range 18–38%). The standard-resection group demonstrated a mean decrease in femoral head perfusion of 7% (range 4–11%). Correlation analysis demonstrated a significant negative correlation (correlation coefficient −0.877; *P* < 0.001; *R* = 0.747). For every 1° that the superior resection margin extended posteriorly, a corresponding 0.88% decrease in femoral head perfusion was found.

The authors concluded that femoral head perfusion is almost fully maintained with arthroscopic osteochondroplasty when the superior resection margin is anterior to the 12 o'clock position. Perfusion is also well maintained if the superior resection margin is extended no more than 10° posterior to 12 o'clock. Further posterior extension correlated with greater decreases in femoral head perfusion.

## CAN T2* MAPPING IMPROVE THE DIAGNOSTIC ACCURACY OF MRI SCAN?

Hesper *et al*. [4] from Dusseldorf, Germany evaluated the diagnostic accuracy of T2*-mapping for detecting acetabular cartilage damage in patients with symptomatic femoroacetabular impingement (FAI).

A total of 29 patients (17 females, 12 males, mean age 35.6 years, mean body mass index 25.1 kg/m^2^, 16 right hips) with symptomatic FAI underwent T2* MRI and subsequent hip arthroscopy. T2* values were obtained by region of interest analysis in seven radially reformatted planes around the femoral neck (anterior, anterior-superior, superior-anterior, superior, superior-posterior, posterior-superior and posterior). Intraoperatively, a modified outerbridge classification was used for assessment of the cartilage status in each region. T2* values and intraoperative data were compared, and sensitivity, specificity, negative predictive values (NPV) and positive predictive values (PPV) as well as the correlation between T2*-mapping and intraoperative findings, were determined. The mean time interval between MRI and arthroscopy was 65.7 ± 48.0 days.

Significantly higher T2* values were noted in arthroscopically normal evaluated cartilage than in regions with cartilage degeneration (mean T2* 25.6 ms vs. 19.9 ms; *P* < 0.001). With the intraoperative findings as a reference, sensitivity, specificity, NPV and PPV were 83.5%, 67.7%, 78.4% and 74.4%, respectively. The correlation between T2*-mapping and intraoperative cartilage status was moderate (ρ = −0.557; *P* < 0.001).

The authors concluded that T2*-mapping enabled analysis of acetabular cartilage with appropriate correlation with intraoperative findings and promising results for sensitivity, specificity, PPV and NPV in this cohort. The results emphasize the value of T2*-mapping for the diagnosis of hip joint cartilage pathologies in symptomatic FAI.

## ARE DELAYS IN DIAGNOSIS ASSOCIATED WITH POORER OUTCOMES IN ADULT HIP DYSPLASIA?

Kennedy *et al*. [5] from Glasgow, UK examined the relationship between delay and diagnosis and outcomes of dysplasia surgery. They identified patients presenting to a hip specialist with a new diagnosis of hip dysplasia. The time taken between patients presenting to their general practitioner and attending the young adult hip clinic was established. Patients were stratified into Early, Moderate and Late Referral groups. Hip and SF-12 questionnaires were completed. Radiographs were graded according to the Tönnis classification system and the outcome following hip specialist review documented.

Fifty-one patients were identified. Mean time from attending a general practitioner to review at the young adult hip clinic was 40.4 months. Lower hip and SF-12 scores, and higher radiological osteoarthritis grades were found in the moderate and late referral groups. A higher proportion of the moderate and late referral group underwent total hip arthroplasty rather than periacetabular osteotomy (PAO).

The authors concluded that delays in referring a patient to a hip specialist are associated with poorer outcomes. They have proposed that pelvic radiographs are requested early by general practitioners to allow prompt diagnosis and referral to a hip specialist.

## DOES PAO INCREASE SPORTS PARTICIPATION COMPARED WITH THR?

Researchers [6] from Fukuoka, Japan compared sports participation and activity levels in Asians after THR and PAO. Multivariate analyses were applied to determine which factors wereassociated with post-operative sports participation and University of California-Los Angeles (UCLA) activity score in (i) 524 THR patients and (ii) 487 acetabular dysplasia patients (295 THR patients and 192 PAO patients). In addition, post-operative sports participation and UCLA score were compared between 62 THR and 62 PAO patients after adjusting for baseline characteristics with propensity score matching.

Sports participation and UCLA score significantly increased after THR (*P* < .001 in both analyses). Preoperative sports participation was the factor most associated with both post-operative sports participation and UCLA score in both 524 THA patients and 487 acetabular dysplasia patients (*P* < .001 in all analyses). Multivariate analysis in 487 acetabular dysplasia patients demonstrated that THR, compared with PAO, was negatively associated with post-operative sports participation (*P* < .001), but not post-operative UCLA score (*P* = .22). THR patients showed significantly lower rate of post-operative sports participation (32.3% and 51.6%, respectively, *P* = .046), but not post-operative UCLA score (5.0 ± 1.6 and 5.2 ± 1.9, respectively, *P* = .47) compared with matched PAO patients.

Both PAO and THA significantly increased sports participation and activity levels with sports participation being significantly higher in patients with PAO. Both multivariate and propensity score-matched analyses showed that post-operative activity levels were comparable between THA and PAO cohorts.

## References

[1] Kester BS, Capogna B, Mahure SA Independent risk factors for revision surgery or conversion to total hip arthroplasty after hip arthroscopy: a review of a large statewide database from 2011 to 2012. Arthroscopy 2018; doi: 10.1016/j.arthro.2017.08.297. Published online 4 January 2018. 10.1016/j.arthro.2017.08.297 29306657

[2] Yu X, Zhang D, Chen X Effectiveness of various hip preservation treatments for non-traumatic osteonecrosis of the femoral head: a network meta-analysis of randomized controlled trials. J Orthop Sci 2017; doi: 10.1016/j.jos.2017.12.004. Published online 29 December 2017. 10.1016/j.jos.2017.12.004 29291916

[3] Lazaro LE, Nawabi DH, Klinger CE Quantitative assessment of femoral head perfusion following arthroscopic femoral osteochondroplasty: a Cadaveric study. J Bone Joint Surg Am 2017; 99: 2094–102. 29257015 10.2106/JBJS.16.01556

[4] Hesper T, Neugroda C, Schleich C T2*-mapping of acetabular cartilage in patients with femoroacetabular impingement at 3 Tesla: comparative analysis with arthroscopic findings. Cartilage 2017; doi: 10.1177/1947603517741168. Published online 10 November 2017. 10.1177/1947603517741168 PMC5871124 29126367

[5] Kennedy JW, Brydone AS, Meek DRM Delays in diagnosis are associated with poorer outcomes in adult hip dysplasia. Scott Med J 2017; 62: 96–100. 28836928 10.1177/0036933017727969

[6] Hara D, Hamai S, Komiyama K Sports participation in patients after total hip arthroplasty vs periacetabular osteotomy: a Propensity Score-Matched Asian Cohort Study. J Arthroplasty 2017; doi: 10.1016/j.arth.2017.08.035. Published online 5 September 2017. 10.1016/j.arth.2017.08.035 28947372

